# Functional Reconstitution of Natural Killer Cells in Allogeneic Hematopoietic Stem Cell Transplantation

**DOI:** 10.3389/fimmu.2016.00144

**Published:** 2016-04-15

**Authors:** Md Ashik Ullah, Geoffrey R. Hill, Siok-Keen Tey

**Affiliations:** ^1^Bone Marrow Transplant Laboratory, QIMR Berghofer Medical Research Institute, Brisbane, QLD, Australia; ^2^Department of Haematology and Bone Marrow Transplantation, Royal Brisbane and Women’s Hospital, Brisbane, QLD, Australia; ^3^School of Medicine, University of Queensland, Herston, QLD, Australia

**Keywords:** NK cell, allogeneic HSCT, NK cell education, memory NK cell, cytomegalovirus

## Abstract

Natural killer (NK) cells are the first lymphocyte population to reconstitute following allogeneic hematopoietic stem cell transplantation (HSCT) and are important in mediating immunity against both leukemia and pathogens. Although NK cell numbers generally reconstitute within a month, the acquisition of mature NK cell phenotype and full functional competency can take 6 months or more, and is influenced by graft composition, concurrent pharmacologic immunosuppression, graft-versus-host disease, and other clinical factors. In addition, cytomegalovirus infection and reactivation have a dominant effect on NK cell memory imprinting following allogeneic HSCT just as it does in healthy individuals. Our understanding of NK cell education and licensing has evolved in the years since the “missing self” hypothesis for NK-mediated graft-versus-leukemia effect was first put forward. For example, we now know that NK cell “re-education” can occur, and that unlicensed NK cells can be more protective than licensed NK cells in certain settings, thus raising new questions about how best to harness graft-versus-leukemia effect. Here, we review current understanding of the functional reconstitution of NK cells and NK cell education following allogeneic HSCT, highlighting a conceptual framework for future research.

## Background

Allogeneic hematopoietic stem cell transplantation (HSCT) can be curative of otherwise incurable leukemia through its ability to mediate an immunological graft-versus-leukemia effect. Its main limitations are graft-versus-host disease (GVHD), infections, and leukemia relapse, all of which are critically dependent on immune reconstitution. Natural killer (NK) cells are well-established mediators of anti-leukemic and anti-viral responses (1). Donor NK cells can also attenuate GVHD, possibly by lysing alloreactive donor T cells and recipient antigen-presenting cells (2–5). There is much interest surrounding the importance or otherwise of NK cells on clinical outcome, particularly in regard to NK cell-mediated GVL effects (5–8). In this review, we will present the current state of knowledge on the functional reconstitution of NK cells to provide a framework to the debate.

## Kinetics of NK Cell Reconstitution

Natural Killer cells are the first donor-derived lymphocyte population to reconstitute numerically following allogeneic HSCT. Normal NK cell numbers are generally observed within the first month post-transplant irrespective of the graft source: bone marrow (9), granulocyte colony-stimulating factor (G-CSF)-mobilized peripheral blood stem cell (PBSC) (9–11), or umbilical cord blood (12–14). It is generally thought that the reconstituting NK cells are primarily derived from the differentiation and maturation of progenitor cells rather than the expansion of mature NK cells within the graft. This concept is supported by two observations. First, the rate of NK cell reconstitution is largely independent of the type of graft and its NK cell content: side-by-side comparisons have found similar reconstitution kinetics following unmanipulated PBSC transplant, CD34^+^-selected PBSC transplant, and bone marrow transplant despite log-fold differences in NK cell content (median 20–70 × 10^6^/kg, 0.2–0.7 × 10^6^/kg, and 5–7 × 10^6^/kg, respectively) (9, 15–17), Second, the early reconstituting NK cells have an immature CD56^bright^ phenotype and do not acquire the predominantly CD56^dim^ donor NK phenotype for several months (17–19). Although NK cell development from progenitors is likely dominant, the *in vivo* expansion of transferred NK cells can also contribute. In a comparison of two different methods of T-cell depletion (CD3/CD19-depletion versus CD34-selection), NK cell reconstitution and acquisition of mature NK cell phenotype were more rapid in recipients of CD3/CD19-depleted grafts, which contained 3-log more mature NK cells than CD34-selected grafts (20). The impact of T cells on NK cell reconstitution is difficult to cleanly define as it is also linked to the use of post-graft immunosuppressive therapy. In haploidentical transplantation using extensively T-cell-depleted graft without post-transplant immunosuppression, NK cell reconstitution is particularly brisk (8) but in other settings where cyclosporine-based immunosuppression is used in both T-cell-deplete and T-cell-replete arms, the reconstitution of NK cell numbers was generally found to be similar between the groups (15, 17, 18).

## Acquisition of NK Cell Functionality

Although NK cells reconstitute numbers by around 1 month post-transplant, they take several months to acquire the immunophenotypic and functional characteristics found in healthy donors. CD56^bright^ NK cells, which are the precursors of CD56^dim^ NK cells (21), account for 40–50% of the NK cells in the first 3 months post-transplant as compared to only 5–10% in healthy donors (17, 19, 22–25). These early reconstituting NK cells also express higher levels of the inhibitory receptor, NKG2A, at around 90% compared to around 50% in healthy donors (17, 22–25). During NK maturation, the CD56^dim^ NK cells lose NKG2A expression and express the activating NKG2C receptor, killer cell inhibitory immunoglobulin-like receptors (KIRs), and CD57 (26, 27). The acquisition of full donor surface phenotype takes 3–6 months, sometimes longer (17, 24–26, 28). Full NK cell functionality is similarly not achieved for at least 6 months post-transplant (17, 24, 29). In healthy individuals, CD56^bright^ NK cells are adapted to produce cytokines, particularly interferon-γ (IFN-γ) and tumor necrosis factor (TNF), whereas CD56^dim^ NK cells are enriched for perforin and granzymes, and thus adapted for cytotoxicity (30, 31). Following allogeneic HSCT, however, there is a dissociation between the recovery of cytokine production and cytotoxic function (29). Despite the high proportion of CD56^bright^ NK cells in the first 6 months post-transplant, IFN-γ production in response to the MHC class I-deficient K562 cell line or primary acute myeloid leukemia cells is more severely and consistently impaired than NK cell degranulation and cytotoxicity (24, 27, 29). This somewhat contradictory finding is nonetheless consistent with the reduced expression of T-bet, a key inducer of IFN-γ production (32), at all stages of NK cell differentiation post-transplant (27). Furthermore, NK cell expression of T-cell immunoglobulin and mucin-containing domain-3 (Tim-3) is also lower post-transplant (33). In healthy individuals, Tim-3 is expressed on nearly all mature CD56^dim^ NK cells and a majority of immature CD56^bright^ NK cells (33, 34). It is upregulated by IL-15 or IL-12 and IL-18 *in vitro* (33, 34), and has been shown to both enhance IFN-γ secretion (33) and suppress cytotoxicity (34). As the level of Tim-3 expression at 3–6 months post-transplant is only half that of healthy controls, this may partly account for the discordant recovery of cytokine production and cytotoxic function (29).

The influence of graft T cell content on NK cell development and function is of clinical interest because the NK cell-mediated GVL effect is most evident in T-cell-depleted transplantation (5–8). While T-cell graft content does not have a significant influence on the numerical reconstitution of NK cells (15, 17, 18), there is a general trend towards enhanced functional NK cell maturation in T-cell-replete versus T-cell-deplete transplants, which is contrary to the relative importance of NK cells in T-cell-deplete transplants. In a study comparing HLA-matched T-cell-replete transplant with immunosuppression versus HLA-partially matched T-cell-deplete transplant without immunosuppression, target cell-induced IFN-γ secretion and degranulation were relatively attenuated in the T-cell-deplete group (29). This is consistent with an earlier study by the same group that found that NK cells in partially T-cell-deplete transplants had attenuated IFN-γ production compared to T-cell-replete transplants, with a similar proportion in both groups receiving cyclosporin A for GVHD prophylaxis (70 versus 81%) (18). Similarly, in a study comparing partial T-cell-depleted transplant (median 54 × 10^4^ T cells/kg) versus extensive T-cell-depleted transplant (median 3.7 × 10^4^ T cells/kg), with neither group receiving post-transplant immunosuppressive therapy, the reconstituting NK cells in the extensively T-cell-depleted group had higher proportions of CD56^bright^ and NKG2A^+^ immature NK cells and diminished cytotoxicity, although IFN-γ secretion was enhanced (19, 22). The mechanism by which T cells facilitate NK cell functional maturation is unclear: it may include the direct activation of CD56^bright^ NK cells by T cell-derived IL-2 (35), or indirectly through IL-12 and IL-18 produced by activated macrophages during acute GVHD (36), and this effect was observed irrespective of the use of post-transplant immunosuppression. It is difficult to isolate the effect of pharmaceutical immunosuppression on NK cell reconsitution because it is tightly linked to the graft T-cell content and subsequent risk of GVHD, both of which can influence NK cell reconstitution. Cyclosporin A does not have any impact on NK cell function in short-term cultures (37) but it has been shown to suppress the *in vitro* proliferation of NK cells, especially the CD56^dim^CD16^+^KIR^+^ NK cells, resulting in a relative increase in the number of immature CD56^bright^CD16^−^KIR^−^ NK cells (38). Hence, cyclosporin A can have a direct effect on NK cells in addition to any indirect effect through modulation of GVHD although more studies, including *in vivo* studies, will be required.

## NK Cell Education Following Allogeneic HSCT

Natural killer cells sense and respond to cellular transformation, stress, and infection via an array of germ-line encoded activating and inhibitory receptors (39). The inhibitory receptors recognizing self-MHC class I are considered the predominant mediators of self-tolerance and the engagement of these receptors with their cognate MHC during NK cell development results in “licensed” NK cells that have functional competency, whereas failure of receptor engagement results in hyporesponsiveness (40–42). In HLA-mismatched transplantation where there is a mismatch in the inhibitory KIR-ligands located on HLA-B and HLA-C loci, there is a potential for donor NK cells that are licensed through the non-shared donor HLA to recognize and attack recipient leukemic cells that lack the cognate inhibitory HLA ligand. This “missing self” NK alloreactivity can be very potent and is associated with decreased risk of relapse in T-cell-depleted haploidentical transplantation for acute myeloid leukemia (5, 6, 43). The importance of MHC class I-mediated NK cell licensing is, however, not entirely clear-cut, particularly in allogeneic HSCT that are HLA-matched or HLA-mismatched but KIR-ligand matched. In both healthy donors and patients post-transplant, there is a hierarchy of target cell-induced NK cell degranulation response: (i) NKG2A^−^KIR^−^ and NKG2A^−^Non-self-KIR^+^ NK cells are hyporesponsive (where non-self KIR recognizes an HLA ligand that is not expressed by the individual), (ii) NKG2A^+^KIR^−^ NK cells and NKG2A^−^Self-KIR^+^ NK cells have similar degrees of responsiveness (where self-KIR is a KIR that recognizes a self HLA ligand), and (iii) NKG2A^+^Self-KIR^+^ NK cells have the highest level of responsiveness (17, 29, 44). Hence, NKG2A have a role in NK cell education post-transplant that is additive to that of inhibitory KIRs. Since KIR expression is reduced for at least 3–6 months post-transplant (17–19, 22, 23, 44), NK cell degranulation response during this time is dominated by NKG2A^+^KIR^−^ NK cells rather than KIR^+^ NK cells (17). The extent to which these NKG2A^+^KIR^−^ NK cells can mediate GVL effect is likely context dependent. The ligand for NKG2A, HLA-E, is often expressed on leukemic cells, and its immune evasive capacity is underscored by the demonstration that antibodies against NKG2A can enhance NK cell-mediated lysis of leukemic cells both *in vitro* and *in vivo* (22, 45). However, not all leukemic cells express HLA-E and other studies have shown relatively low levels of HLA-E expression on primary leukemic blasts (19, 46) and since HLA-E is expressed in complex with a signal peptide from certain MHC class I molecules, its expression is also low in MHC class I-deficient blasts, and in this regard, the HLA-E/NKG2A interaction can be considered to be analogous to that of MHC class I/inhibitory KIRs.

**Figure 1:**
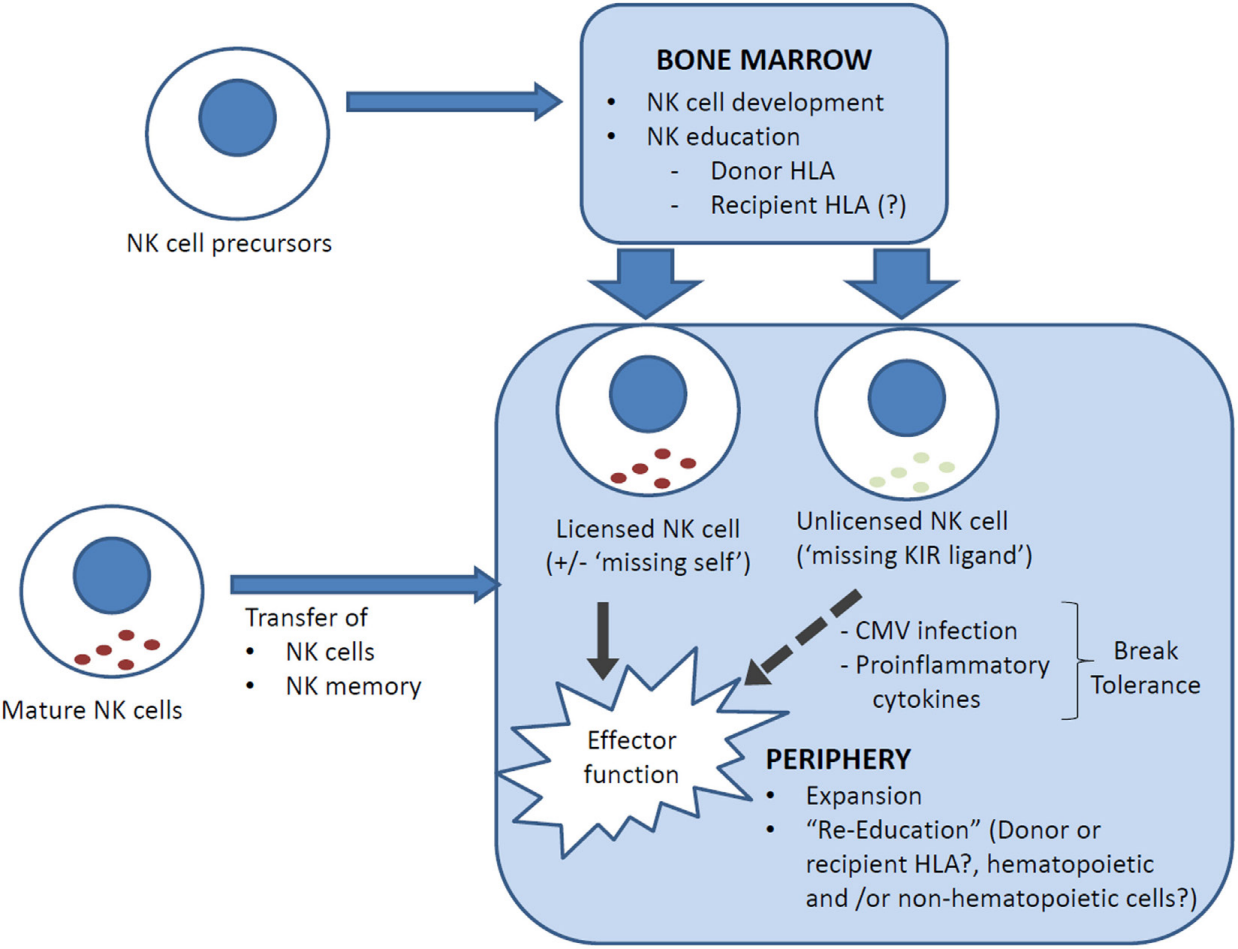
**NK cell reconstitution and education following allogeneic HSCT**. Reconstituting NK cells can be derived from (i) NK cell precursors, ranging from hematopoietic stem cells through to common lymphoid progenitors, that differentiate into NK cells in the bone marrow, and (ii) transferred mature NK cells, which carry with them a mature NK phenotype and NK memory. NK cells are educated in the bone marrow but can be “re-educated” in the periphery. It is uncertain whether recipient cells (generally non-hematopoietic only post transplant) are involved in NK cell education. In HLA-mismatched transplants, NK cells that are licensed by inhibitory KIRs that recognize a ligand (HLA) that is expressed only by the donor and not the recipient may lyse recipient cells (“missing self”) and contribute to the GVL effect. Conversely, an NK cell that has not encountered a cognate MHC class I for its inhibitory KIR(s) is “unlicensed” and hyporesponsive, but can acquire functional competency when stimulated by pro-inflammatory cytokines, for example, in the setting of CMV reactivation.

Can allogeneic HSCT break NK tolerance? In the above studies, drawn from both T-cell-replete and T-cell-deplete transplants, with or without cyclosporine A, NK cell expression of at least one inhibitory receptor remained necessary for NK cell functional competency as NKG2A^−^ NK cells that were KIR^−^ or expressed only non-self KIR remained hyporesponsive (17, 29, 44). There is, however, evidence that NK cell tolerance can be broken post-transplant. In one study, unlicensed NK cells that express single non-self inhibitory KIR were found to have increased cytokine secretion and cytotoxicity at 3–6 months post-transplant compared to their respective donors, and this effect was independent of NKG2A expression (47). The mechanism underpinning this is unclear but there are clues from murine models. In mice, NK cell function can be restored *in vitro* with IL-2 or IL-12 + IL-18, or strong stimulation via activating receptors (41, 48, 49). *In vivo*, NK cell tolerance can be broken by infection with *Listeria monocytogenes* and murine cytomegalovirus (MCMV) (50–52). Indeed, NK cell activation early post MCMV infection is dependent on pro-inflammatory cytokines and independent of activating receptor ligation, and licensed and unlicensed NK cells were similarly activated and produced similar levels of IFN-γ and granzyme B (52, 53). Since CMV reactivation is a common complication of allogeneic HSCT, it is possible that a similar mechanism underpinned the clinical observation. Unlicensed NK cells that break tolerance are not merely bystanders but can have specific protective function. In the MCMV model, unlicensed NK cells proliferated more robustly than licensed NK cells and were more effective in controlling MCMV infection because, unlike licensed NK cells, they were not inhibited by MHC class I expression on target cells (52). Similarly, unlicensed NK cells have been shown to be the primary mediators of antibody-dependent cell-mediated cytotoxicity during monoclonal antibody treatment for neuroblastoma (54). In HLA-matched and mismatched allogeneic HSCT, the risk of acute myeloid leukemia relapse is lower in patients who lack one or more HLA ligands to inhibitory KIRs (“missing KIR-ligand” hypothesis) (55, 56), which further supports the importance of unlicensed NK cells as a mediator of GVL effects (Figure 1).

Since the first reports on the protective effect of KIR-ligand mismatching on leukemia relapse more than 10 years ago (5, 57), our understanding of NK cell education has evolved and the “missing self” hypothesis is more complex than it initially seemed. It is now known that mature NK cells can undergo “re-education” following transfer into a different MHC environment; thus, mature responsive NK cells from wild-type mice become hyporesponsive when transferred to MHC class I-deficient mice and vice versa (58, 59). Furthermore, the education process requires the MHC class I to be expressed on all or most cells, or hyporesponsiveness is dominantly induced (41); and both non-hematopoietic as well as hematopoietic cells may be involved in NK cell education (58, 60). These new insights suggest that recipient MHC class I-mediated NK cell education may diminish the anti-leukemic effect of “missing self” NK alloreactivity, and are consistent with the observation that alloreactive donor NK cells were detectable mainly in the first 3 months post-transplant (57).

## Impact of CMV on NK Reconstitution

Cytomegalovirus reactivation is a common and life-threatening complication following allogeneic HSCT. In healthy individuals, CMV serostatus accounts for a significant proportion of the variability in NK cell immunity (61–63). Similarly, in allogeneic HSCT, CMV reactivation influences the frequency, phenotype, function, and/or repertoire of the reconstituting immune system (64–70). Interestingly, CMV reactivation is also associated with lower risks of leukemia relapse (71, 72), and understanding its influence on the immune landscape may provide insight into new therapeutic approaches to enhance the GVL effect.

The concept that NK cells can acquire immunological memory with features that are classically associated with T and B cell responses is largely established by studying CMV infection in mice (73, 74) and humans (61, 62, 75–78). In mice, MCMV infection induces the expansion of NK cells that express the activating receptor, Ly49H, which recognizes the viral protein m157 on the surface of infected cells (74, 79–81). Ly49H^+^ NK cells undergo marked expansion, followed by contraction, and establishment of a long-lived memory population that mounts a more effective protective response than naive NK cells against MCMV but not heterologous infections (73, 74, 76). In humans, CMV infection induces an expansion of NK cells that express NKG2C (75, 76, 82, 83), an activating killer lectin-like receptor that binds HLA-E, which is upregulated by CMV UL40 protein (84, 85). These NKG2C^+^ memory NK cells are CD56^dim^CD57^bright^ and have a highly differentiated phenotype in regard to cytokine secretion and degranulation (75). They are preferentially negative for NKG2A (76, 82, 83) and are biased toward the expression of self-specific “licensing” inhibitory KIRs: KIR2DL3 in HLA-C1^+^ individuals and KIR2DL1 in HLA-C2^+^ individuals (76).

Similarly, in allogeneic HSCT, CMV reactivation is followed by an increase in the proportion of NKG2C^+^ NK cells within 2–4 weeks, which persists for at least a year (86, 87). These NK cells also have a more mature NKG2C^+^CD57^+^ phenotype and are predominantly KIR^+^, especially for inhibitory self-KIRs (KIR2DL2/3), and secrete more IFN-γ than NKG2C^−^ NK cells (67, 87). CMV-seropositive recipients without overt CMV reactivation have an NK memory phenotype intermediate between patients with CMV reactivation and CMV-seronegative recipients, but only if they received a sibling allograft and not umbilical cord blood, which is generally considered to be CMV naive (67). This observation would suggest that donor NK memory could be transferrable. In support of this hypothesis, the same group had previously demonstrated that the NKG2C^+^ NK cells that emerged following CMV reactivation had increased levels of IFN-γ production when the donor was CMV seropositive rather than CMV seronegative (88). Furthermore, NK memory is undoubtedly transplantable in experimental mouse systems (74). The contribution of NK memory transfer in clinical allogeneic HSCT remains to be ascertained as NK reconstitution is primarily attributed to new NK cells generated from hematopoietic precursors rather than the expansion of NK cells from the graft, although this too remains to be conclusively demonstrated. NKG2C is not the only activating receptor relevant to CMV infection. CMV infection in healthy individuals also expands NK cells that express other activating receptors, including the activating KIRs: KIR2DS2 and KIR2DS4 (76). In allogeneic HSCT, recipients of umbilical cord blood transplant from donors with homozygous deletion of *NKG2C*, which represents 4% of the healthy population, have increased numbers of CD56^dim^NKG2A^−^ActivatingKIR^+^ NK cells following CMV reactivation (69).

More recently, a distinct subset of FcRγ (also known as FcϵRIγ)-deficient NK cells has been identified in CMV-seropositive individuals (89, 90). They are predominantly, but not exclusively, NKG2C^+^, and respond poorly to CMV-infected lung fibroblasts, but display enhanced antibody-dependent expansion, degranulation, and cytokine secretion (61, 90). FcRγ-deficient NK cells can be detected in some patients at 6–12 months after umbilical cord blood transplantation, but only if they had prior CMV reactivation (62). This memory-like FcRγ^−^ NK phenotype is the result of epigenetic modification with hypermethylation of the *FCER1G* promoter (62). Epigenetic silencing also results in a deficiency of the cell signaling proteins SYK and EAT-2, and transcription factors PLZF and IKZF2, within this population (61, 62). The significance of this newly described NK population in allogeneic HSCT remains to be investigated.

## Impact of GVHD on NK Reconstitution

Natural killer cell numbers were found to be lower in patients with acute and chronic GVHD (16, 91), but it is not known if these were casually related given the confounding effects of T cells, immunosuppression, and other clinical variables. On the other hand, acute GVHD is associated with the secretion of pro-inflammatory cytokines, for example, IL-12 and IL-18 (36), which are known to promote NK cell functional maturation. Acute GVHD is also associated with elevated levels of soluble ST2 (92), which serves as a decoy receptor to modulate the IL-33/ST2 axis (93, 94). This raises the possibility of effect on NK cells as IL-33/ST2 axis augments NK cell production of IFN-γ in response to IL-12 (95), and is important in MCMV-specific expansion of naive and memory Ly49H^+^ NK cells (96). At present, all these concepts remain speculative and require further investigation.

## Innate Lymphoid Cells

The lineage marker-negative innate lymphoid cells (ILCs) are a recently identified family of lymphoid cells that are preferentially located at barrier surfaces and can rapidly secrete immunoregulatory cytokines that correspond to the T_H_1, T_H_2, or T_H_17/T_H_22 immune response (97, 98). Their role in allogeneic HSCT is gradually being elucidated and it has been recently shown that recipient-derived intestinal ILCs are important in mediating protection from gut GVHD (99, 100). However, the nature and role of donor-derived ILC reconstitution remains largely unknown at present (101).

## Conclusion

Natural killer cells reconstitute rapidly after HSCT but the delayed acquisition of a mature phenotype and functional competency argues for strategies to enhance functional NK cell reconstitution. These strategies can include adoptive transfer (102–104), with or without *ex vivo* expansion and cytokine activation (105, 106), graft engineering (20), donor selection according to KIR haplotype, and exogenous cytokine administration (107). Key unanswered questions relevant to optimizing NK-mediated anti-leukemic and anti-viral immunity include: what are the desired phenotypic characteristics of NK cells in this regard? What are the relative roles of unlicensed and licensed NK cells? Does NK cell memory contribute to long-term tumor immune surveillance? How are NK cells educated in HLA-mismatched transplantation and does this change over time? Additionally, what is the nature of ILC reconstitution post allogeneic HSCT? The answers to these questions are important in improving transplant outcome and further experimental and clinical studies are needed.

## Author Contributions

MU wrote the manuscript. GH conceptualized and edited the manuscript. S-KT conceptualized, wrote, and edited the manuscript.

## Conflict of Interest Statement

The authors declare that the research was conducted in the absence of any commercial or financial relationships that could be construed as a potential conflict of interest.
